# Polymorphisms in Autophagy Genes Are Associated with Paget Disease of Bone

**DOI:** 10.1371/journal.pone.0128984

**Published:** 2015-06-01

**Authors:** Ricardo Usategui-Martín, Judith García-Aparicio, Luis Corral-Gudino, Ismael Calero-Paniagua, Javier Del Pino-Montes, Rogelio González Sarmiento

**Affiliations:** 1 Unidad de Medicina Molecular-IBSAL, Departamento de Medicina, Universidad de Salamanca-Hospital Universitario de Salamanca-CSIC, Salamanca, España; 2 Servicio de Medicina interna-IBSAL, Hospital Universitario de Salamanca Universidad de Salamanca CSIC, Salamanca, España; 3 Servicio de Reumatología-IBSAL, Hospital Universitario de Salamanca-Universidad de Salamanca-CSIC, Salamanca, España; 4 Instituto de Biología Molecular y Celular del Cáncer (IBMCC), Universidad de Salamanca-CSIC, Salamanca, España; IISER-TVM, INDIA

## Abstract

Paget disease of bone (PDB) is a focal bone disorder affecting the skeleton segmentally. The main alteration resides in osteoclasts that increase in size, number and activity. Many osteoclasts have cytoplasmic inclusions that have been associated with protein aggregates, increasing the evidences of a possible deregulation of autophagy in the development of the PDB. Autophagy starts with encapsulation of the target into a double-membrane-bound structure called an “autophagosome.” It has been reported that at least 18 ATG genes (autophagy-related genes) are involved in autophagosome formation. We have studied the distribution of genotypes of the ATG2B rs3759601, ATG16L1 rs2241880, ATG10 rs1864183 and ATG5 rs2245214 polymorphisms in a Spanish cohort of subjects with PDB and compared with healthy subjects. Our results show that being a carrier of the C allele of the ATG16L1 rs2241880 and the G allele of ATG5 rs2245214 polymorphisms were associated with an increased risk of developing PDB, whereas being a carrier of the T allele of ATG10 rs1864183 polymorphism decreased the risk of suffering the disease in our series. This is the first report that shows an association between autophagy and Paget Disease of Bone and requires further confirmation in other series.

## Introduction

Paget's disease of bone (PDB) is a focal disorder of bone that affects segmentally the skeleton. The main alteration resides in osteoclasts that increase in size, number and activity. A change occurs in bone remodelling consisting in an increase in bone resorption followed by an excessive bone formation that results in a variegated and anarchic bone structure that alters the mechanical properties [1,2]. Currently, PDB is the most common metabolic bone disease after osteoporosis [3].

Two are the etiopathogenic hypotheses that attempt to explain the origin of the PDB: the influence of environmental factors and the existence of genetic factors [1,2]. Several environmental agents have been postulated as possible etiologic factors in the PDB. The most frequently environmental agents implicated are the infectious agents, especially viruses due to the fact that in the cytoplasm of many osteoclasts were observed structures that have been associated with viral nucleocapsids. [1,4]. It has been proposed also that these structures could be protein aggregates similar to those seen in neurodegenerative diseases [1,5,6], increasing the evidences of a possible deregulation of autophagy in the development of the PDB [1,7].

Between 20–40% of patients with a positive family history of the disease and 5–10% of sporadic patients are carriers of a mutation in the sequestosome1 gene (SQSTM1) which encodes the p62 protein [8,9]. The p62 protein plays an important role in cellular signals crossroads related with the activation of the NF-kB pathway [10] and autophagy [11,12]. The p62 protein has a size of 434 aminoacids (62kDa) and it consists in different domains. Between the two PEST domains is a LIR domain, which interacts with the LC3 protein that allows the anchoring of p62 to the autophagosome, facilitating the degradation of ubiquitinated proteins [13,14].

Autophagy is a catabolic process responsible for the degradation of damaged organelles, cytoplasmic proteins and protein aggregates. It starts with the formation of the “autophagosome”, a double membrane structure in which the target is encapsulated. “Autophagosomes” fuse with lysosomes containing hydrolases that are responsible for degrading its contents [15–17]. Molecular studies in *saccharomyces cerevisiae* have identified at least 18 ATG genes (autophagy-related genes) involved in autophagosome formation [16–18].

There are several evidences that suggest that the development of PDB may be related to a deregulation of autophagy. Genetic variants of several genes involved in the process of autophagy such as SQSTM1, VCP and OPTN have been linked with PDB [1]. Moreover, it has been recently reported that mice with SQSTM1 mutation and that develop a PDB phenotype have increased expression of genes involved in autophagy such as SQSTM1, ATG5 and LC3 [1,7]. In patients with PDB, the p62 protein is over-expressed regardless of the SQSTM1 mutation status, suggesting that there could be a decrease in autophagy [19,20].

The aim of our study was to characterize whether polymorphisms in genes involved in autophagy would modify the risk of developing PDB. We have studied ATG2B rs3759601, ATG16L1 rs2241880, ATG10 rs1864183 and ATG5 rs2245214 polymorphisms to evaluate their putative role in the susceptibility of suffering PDB in a cohort of Spanish patients.

## Materials and Methods

### Subjects

We have studied 238 patients with PDB from the province of Salamanca, Spain. According to the prevalence of PDB [3] we calculated that a series of 152 patients would be representative of our PDB population. In the familial cases we have included only one affected patient of each family. Patients were recruited in the Metabolic Bone Unit at the University Hospital of Salamanca between January 1990 and February 2014. As a control group, 264 sex-matched healthy subjects over 40 years old without previous history of PDB were recruited to the same hospital during the same period of time. To confirm that the healthy subjects had not a silent PDB, we tested alkaline phosphatase levels and performed bone radiography to exclude bone affection. From each patient were collected clinical variables such as gender, age of diagnosis, family history, number of affected bones, presence of fractures, involvement of the skull and cranial nerve involvement. The study was approved by the local Ethics Committees of the University Hospital of Salamanca (Salamanca, Spain). The patients have signed an informed consent.

### DNA isolation and polymorphism genotyping

Genomic DNA was extracted from peripheral blood by standard phenol/chloroform procedure. Genotyping of ATG2B rs3759601, ATG16L1 rs2241880, ATG10 rs1864183 and ATG5 rs2245214 polymorphisms was performed using TaqMan 5´-exonuclease allelic discrimination assays (Table 1) that contain sequence-specific forward and reverse primers to amplify the polymorphic sequences and two probes labeled with VIC and FAM dyes to detect both alleles of each polymorphism [21]. PCR reactions were carried out using TaqMan universal PCR Maxter Mix following instructions in a Step-One Plus Real-time PCR system. To assess reproducibility, a random selected 5% of the samples were re-genotyped, all of these genotypes matched with genotypes initially designated. We have selected polymorphisms from ATG genes involved in autophagosome generation that have been previously reported in the literature. Initially, we selected non-synonym polymorphisms with a population frequency of the minor allele higher than 10% in Caucasian population and that were located in sequences highly conserved throughout the evolution. ATG2B rs3759601, ATG16L1 rs2241880 and ATG10 rs1864183 polymorphisms are missense mutations, whereas ATG5 rs2245214 polymorphism is intronic, but we selected this polymorphism because it was previously reported in the literature, the frequency of the minor allele was higher than 10% in Caucasians and because this polymorphism is associated with the lost of a recognition sites for SRp40 protein that is involved in mRNA splicing.

**Table 1:** Autophagy polymorphisms analysed in the study.

Gene	SNP ID	Base change	SNP	Chr location	Assay ID^a^	HWE^b^
ATG2B	rs3759601	C>G	Q1383E	14	c_9690166_10	>0.05
ATG16L1	rs2241880	T>C	T300A	2	c_9095577_20	>0.05
ATG10	rs1864183	C>T	T212M	5	c_11953871_20	>0.05
ATG5	rs2245214	C>G	Intronic	6	c_3001905_20	>0.05

^a^All the assays were commercially

^b^ HWE: Hardy-Weinberg equilibrium in control group

### Statistical analyses

Healthy subjects group were tested for conformity to the Hardy-Weinberg equilibrium using chi-squared test for each polymorphism. Odds ratios (ORs) and 95% confidence intervals (95% CIs) were estimated for each polymorphic variant using unconditional logistic regression models to evaluate the association with PDB risk. These statistical analyses were performed using SPSS software. For the analysis, differences with a p-value<0.05 were considered as statistically significant.

## Results

A total of 238 PDB patients and 264 healthy subjects were analysed. The clinical variables for each patient are summarized in Table 2. The distribution of genotypes of ATG2B rs3759601, ATG10 rs1864183, ATG16L1 rs2241880 and ATG5 rs2245214 polymorphisms in control samples were in Hardy-Weinberg equilibrium (Table 1).

**Table 2:** Clinical characteristics of PDB patients.

		PDB PATIENTS
Gender	Man	132
	Woman	106
Age of diagnosis	Over 60 years	188
	Under 60 years	50
Family history	Sporadic	213
	Familial	25
Number of affected bones	Fewer than three	177
	More than three	61
Presence of fractures	Yes	17
	No	221
Involvement of the skull	Yes	90
	No	148
Cranial nerve involvement	Yes	33
	No	205

The genotypic frequencies and the result of the association analysis resulting from the study ATG2B rs3759601, ATG10 rs1864183, ATG16L1 rs2241880 and ATG5 rs2245214 polymorphisms in PDB and healthy subjects are summarized in Table 3. No significant differences were found in genotypic distribution for ATG2B rs3759601 polymorphism between PDB group and healthy subjects. However, we found statistically significant differences in genotypic distribution for ATG16L1 rs2241880, ATG10 rs1864183 and ATG5 rs2245214 polymorphisms. In the case of ATG10 rs1864183 polymorphism being a carrier of the variant allele T decreased the risk of developing PDB. Homozygous CC genotype of the ATG16L1 rs2241880 polymorphism was associated with increased risk of developing PDB. In the case of ATG5 rs2245214 polymorphism, codominance recessive analysis shows that be carriers of the G allele (CG+GG genotypes) confers an increased risk of developing PDB (Table 3).

**Table 3:** Genotypic frequencies of autophagy genes polymorphisms among cases and controls and the association with PDB risk.

SNP	Genotype	PDB Patients	Controls	p-value	OR (95% IC)
ATG2B rs3759601	CC	90 (37.8%)	110 (41.7%)		
	CG	120 (50.5%)	115 (43.6%)	0.276	
	GG	28 (11.8%)	39 (14.8%)		
	CC+CG	210 (88.2%)	225 (85.2%)	/	
	GG	28 (11.8%)	39 (14.8%)	0.322	
	CC	90 (37.8%)	110 (41.7%)	/	
	CG+GG	148 (62.2%)	154 (58.3%)	0.379	
ATG10 rs1864183	CC	100 (42%)	68 (25.8%)	/	1.00
	CT	107 (45%)	151(57.2%)	**<0.001**	0.48(0.32–0.71)
	TT	31 (13%)	45(17.0%)	**0.007**	0.46 (0.27–0.81)
	CC+CT	207 (87.0%)	219 (83.0%)	/	
	TT	31 (13.0%)	45 (17.0%)	0.211	
	CC	100 (42%)	68 (25.8%)	/	1.00
	CT+TT	138 (58%)	196 (74.2%)	**<0.001**	0.47 (0.32–0.69)
ATG16L1 rs2241880	TT	40 (16.8%)	63 (23.9%)	/	1.00
	TC	110 (46.2%)	138 (53.3%)	0.342	1.25 (0.78–2.00)
	CC	88 (37.0%)	63 (23.9%)	**0.003**	2.20 (1.31–3.66)
	TT+TC	150 (63%)	201 (76.1%)	/	1.00
	CC	88 (37%)	63 (23.9%)	**0.001**	1.87 (1.27–2.75)
	TT	40 (16.8%)	63 (23.9%)	/	
	TC+CC	198 (83.2%)	201 (76.1%)	0.052	
ATG5 rs2245214	CC	74 (31.1%)	106 (40.2%)		
	CG	128 (53.8%)	127 (48.1%)	0.094	
	GG	36 (13.1%)	31 (11.7%)		
	CC+CG	202 (84.9%)	233 (88.3%)	/	
	GG	36 (15.1%)	31 (11.7%)	0.267	
	CC	74 (31.1%)	106 (40.2%)	/	1.00
	CG+GG	164 (68.9%)	158 (59.8%)	**0.035**	1.48 (1.02–2.15)

Significant p-values are represented in bold.

The distribution of allelic frequencies for ATG2B rs3759601, ATG10 rs1864183, ATG16L1 rs2241880 and ATG5 rs2245214 polymorphisms are showed in Table 4. No significant differences were found in the allelic distribution for ATG2B rs3759601 polymorphism between PDB group and healthy subjects. However, we found statistically significant differences in allelic distribution for ATG16L1 rs2241880, ATG10 rs1864183 and ATG5 rs2245214 polymorphisms between PDB patients and controls. In the case of ATG10 rs1864183 polymorphism being a carrier of allele T confers a decreased risk of developing PDB. Allele C of ATG16L1 rs2241880 polymorphism confers an increased risk of developing PDB. In the ATG5 rs2245214 polymorphism, allele G confers an increased risk of developing the disease (Table 4). Moreover, carrying the allele G and allele C of ATG5 rs2245214 and ATG16L1 rs2241880 polymorphisms increases the risk of developing PDB (Table 5).

**Table 4:** Allele frequencies of autophagy gene polymorphisms among cases and controls and the association with PDB risk.

SNP	Allele	PDB Patients	Controls	p-value	OR (95% IC)
ATG2B rs3759601	C	300 (63.0%)	335 (49.7%)		
	G	176 (37.0%)	193 (49.0%)	0.890	
ATG10 rs1864183	C	307 (64.5%)	287 (54.4%)	/	1.00
	T	169 (35.5%)	241 (45.6%)	**0.001**	0.65 (0.50–0.84)
ATG16L1 rs2241880	T	190 (39.9%)	264 (50.0%)	/	1.00
	C	286 (60.1%)	264 (50.0%)	**0.001**	1.50 (1.17–1.93)
ATG5 rs2245214	C	276 (58%)	339 (64.2%)	/	1.00
	G	200 (42%)	189 (35.8%)	**0.044**	1.30 (1.10–1.67)

Significant p-values are reprinted in bold.

**Table 5:** Haplotype distribution of ATG5 and ATG16L1 autophagy genes polymorphism among cases and controls and the association with PDB risk.

SNP	Allele	PDB Patients	Controls	p-value	OR (95% IC)
ATG16L1/ATG5	T/C	105 (44.1%)	146 (53.3%)	/	1.00
ATG16L1/ATG5	C/G	133 (53.9%)	118 (44.7%)	**0.013**	1.56 (1.10–2.23)

Significant p-values are reprinted in bold.

No significant differences were found in the analysis of the different clinical forms and the genotypic distributions of the polymorphisms included in our study.

## Discussion

Paget Disease of Bone (PDB) is consequence of an alteration in bone metabolism that increase bone resorption followed by an excessive compensatory bone formation [1]. The etiology of PDB is unknown. Nevertheless, there are several observations that suggest that autophagy may be involved in the development of the disease [1]. Studying ATG2B rs3759601, ATG16L1 rs2241880, ATG10 rs1864183 and ATG5 rs2245214 polymorphisms we attempted to evaluate the putative role of these variables in the susceptibility to PDB. Three polymorphisms are missense and produce amino acid changes (ATG2B rs3759601, ATG16L1 rs2241880, ATG10 rs1864183) and one is an intronic polymorphism (ATG5 rs2245214) (Table 1). We have selected ATG5, ATG10 and ATG16L1 because they code for proteins that form the ATG5-ATG12 conjugation complex along with ATG16L1, whereas ATG2B is necessary for closure of isolation membranes of autophagosomes.

The ATG16L1 gene is a central adaptor required for the formation of autophagosome [22]. ATG16L rs2241880 polymorphism has been associated with risk of developing Crohn disease [23–25]. We observed that in our series being a carrier of the homozygous CC genotype (p.300 Ala/Ala) is associated with an increased risk of developing PDB. It has been demonstrated that amino acids 296–299 in ATG16L1 constitute a caspase cleavage motif and the p. 300 Thr>Ala variant increased ATG16L sensitivity to caspase-3-mediated processing increasing the degradation of the p.300Ala variant of ATG16L1, resulting in diminished of autophagy [25]. It has been reported that decreased autophagy predisposes to suffer PDB [1,7,19,20]. Thus, being a carrier the CC genotype (Ala/Ala) of the ATG16L1 rs2241880 polymorphism represents a risk of developing PDB because the p.300Ala variant of ATG16L1 protein results in diminished autophagy and could increase the risk to suffer PDB in our series [25].

The ATG5-ATG16L1-ATG12 complex determines the sites of autophagosome synthesis regulating the targeting of LC3 to ATG5-ATG12. During autophagy, this complex will be degraded [16,17]. Mice that exhibit a PDB phenotype have increased expression of ATG5 that might indicate a decrease of autophagy in the course of the disease [7]. ATG5 rs2245214 polymorphism has been previously studied in patients with tuberculosis [26] and lupus erythematous [27]. In our study being a carrier of the G allele increases the risk of developing PDB. We speculate with the possibility that carrying the G allele, that is located within an intron, could indirectly causes a decrease of autophagy or it could be linked to another exonic polymorphism not included in our study and therefore could predisposes to suffer PDB in our series.

It has been reported that down-regulation of ATG genes accelerate tumour development because decreased autophagy enhances tumour development [28,29]. In our study, being a carrier of the T allele of ATG10 rs1864183 polymorphism confers a decreased risk of developing PDB, being this association stronger in the group of subjects carrying the homozygous TT genotype. The T allele codes for a Methionine at codon 212 of ATG10. We could hypothesize that p.212Met variant of ATG10 protein increases autophagy decreasing the risk of developing the disease. Recent studies show that TT genotype of ATG10 rs1864183 polymorphism alters IL8 production [26]. It is well known that cytokines are involved in the interactions between the osteogenic cells in normal bone and IL-8 has been related with osteoclastogenesis and bone resorption [30,31]. In a previous report of 172 patients with PDB from Salamanca (Spain) we were unable to find significant differences in genotype or allelic frequencies between PDB patients and healthy subjects for the -251 IL8 polymorphism [32]. It would be of interest to analyse the levels of IL-8 in patients with EOP and relate them to the TT genotype of ATG10 rs1864183 polymorphism.

ATG2B is essential for autophagosome formation and important for regulation of size and distribution of lipid droplets [33]. This is the first study to investigate the ATG2B rs3759601 polymorphism. Our results show that genotype frequencies did not significantly differ between PDB patients and healthy subjects. This could reflect that variations in genotype of the ATG2B rs3759601 polymorphism do not modify autophagy.

We did not find significant differences when we compared the distribution of ATG2B rs3759601, ATG10 rs1864183, ATG16L1 rs2241880 and ATG5 rs2245214 genotypes and the PDB clinical characteristic. Thus, our results suggest that autophagy would be involved in PDB onset but it is not imply in the clinical outcome.

PDB occurs as a consequence of an increase of bone resorption followed by an excessive bone formation. The main alteration resides in osteoclasts that increase in size, number and activity. Many osteoclasts have cytoplasmic inclusions which have been associated with a possible deregulation of autophagy [1]. It has been reported that decreased autophagy increased the risk to suffer PDB, so the cytoplasmic inclusions could correspond with protein aggregates that have not been degraded [1,7,19,20]. Our results show that being a carrier of G allele of the ATG16L1 rs2241880 and G allele of ATG5 rs2245214 polymorphisms is associated with increased risk of developing PDB in a Spanish cohort; whereas being a carrier of the T allele of ATG10 rs1864183 polymorphism decreased the risk of developing the disease. Additional studies in other series of patients will be necessary to validate our findings.
